# Design and Validation of an Instrument to Evaluate the Learning Acquired by Nursing Students from a Brief Tobacco Intervention (BTI-St©)

**DOI:** 10.3390/ijerph16203944

**Published:** 2019-10-16

**Authors:** Antonio Jesús Ramos-Morcillo, César Leal-Costa, Ana Teresa García-Moral, Rafael del-Pino-Casado, María Ruzafa-Martínez

**Affiliations:** 1 Department of Nursing, Faculty of Nursing, University of Murcia, 30100 Espinardo, Spain; ajramos@um.es (A.J.R.-M.); maruzafa@um.es (M.R.-M.); 2 Jaén Nordeste Sanitary District, Regional Ministry of Health of the Andalusian Regional Government, Úbeda, 23400 Jaén, Spain; tec.promocion.salud.ajaene.sspa@juntadeandalucia.es; 3 Department of Nursing, School of Health Sciences, University of Jaén, 23071 Jaén, Spain; rdelpino@ujaen.es

**Keywords:** brief advice, smoking cessation, tobacco sse cessation, validation studies, counseling, criterion referenced tests, tobacco cessation, 5As, student, nursing students

## Abstract

The aim of this study was to design and validate an instrument, based on the WHO 5As+5Rs model, to test the acquisition by nursing students of a brief tobacco intervention (BTI) learning. A validation design of an instrument following the criterion referenced tests model using videos of simulated BTIs in the primary care setting was carried out. The study included 11 experts in smoking prevention/care and 260 second-year nursing students. The study was in two stages: (1) selection and recording of clinical simulations (settings), and (2) test construction. Content was validated by applying the Delphi consensus technique and calculating the Content Validity Ratio (CVR) and Content Validity Index (CVI). A pilot test was conducted for item analysis. Reliability was evaluated as internal consistency (Kuder-Richardson [KR-20]) and test-retest temporal stability (intraclass correlation coefficient [ICC]). Three simulation settings were recorded. An instrument (BTI-St^®^) was developed with 23 items for dichotomous (yes/no) response. CVR was >70% for all items, KR-20 of 0.81–0.88, and ICC between 0.68 and0.73 (*p* < 0.0001). The BTI-St^®^ is a robust and reliable instrument that is easily and rapidly applied. It follows the WHO 5As+5Rs model and offers objective criterion-referenced evaluation of BTI learning in nursing students.

## 1. Introduction

Smoking is a major public health threat that annually claims the lives of around 6 million smokers [1] and is the main avoidable cause of premature death worldwide [2]. It is estimated to be responsible for 71% of lung cancers, 42% of chronic respiratory diseases, and 10% of cardiovascular diseases [3]. The prevalence of smoking remains at over 20% in high-income countries [4], and the WHO has developed a wide-reaching program to eradicate this habit, including the MPOWER anti-smoking package for a brief tobacco intervention (BTI) in primary care [5]. This intervention has proven effective when provided by physicians, nurses, and dentists [6,7,8], can be readily adopted by health care institutions [9], and is considered cost-effective over the long term [10].

However, although simple and effective interventions are available, their application is not assured. Thus, a systematic review showed that only 65% of family physicians asked patients about their tobacco habit and only 63% advised them to quit [11]. A study of 3482 American nurses found that 73% asked about patients’ tobacco consumption and only 62% recommended its cessation [12]. A meta-analysis of 15 studies carried out in the USA, Canada, and four European countries [13] concluded that nurses mainly ask about tobacco consumption and less frequently assist with quitting (5–52%) or arrange a follow-up program with the patient (3–26%). It found that nurses who smoked were 13% less likely to advise their patients to quit and 25% less likely to organize a follow-up of smoking cessation. The training of professionals on this issue is crucial to increase the implementation of brief tobacco interventions (BTIs). A meta-analysis showed that professionals trained in these interventions are more likely to implement anti-tobacco measures [14]. In fact, the WHO called for education on BTIs to be included in the curricula of Schools of Health Science [15].

### Background

Various methods have been used to facilitate BTI learning [16,17]. These are mostly based on the 5As+5Rs model of the US Public Health Service [18] and WHO [19], in which the intervention follows a sequence of five counseling strategies: “Ask” (ask all patients about tobacco use), “Advise” (advise all tobacco users to quit), “Assess” (assess the readiness to quit), “Assist” (assist with quitting), and “Arrange” (arrange follow-up). The “Assist” option includes the 5Rs: “Relevance”, “Risks”, “Rewards”, “Roadblocks”, and “Repetition”. 

Various approaches have been adopted to test the learning acquired after BTI training, including: multiple-choice tests [20], self-reports [21], patient reports with peer evaluation by health care professionals [22], evaluation by experts using videotaped objective structured clinical examinations (VOSCEs) [16], and evaluation by experts based on direct observations of real encounters and objective structured clinical examinations (OSCEs) [23]. The selection of the most appropriate model depends on the purpose of the evaluation [23].

Criterion-referenced tests (CRTs) have been little used in this context, but their characteristics and objectives make them of special interest. They serve to monitor the progress of students, objectively confirm their performance, diagnose learning deficits, and evaluate educational programs [24]. No standardized and validated CRT is yet available to assess the acquisition of BIT learning; therefore, the objective of this study was to design and validate a CRT based on the 5As+5Rs model in a population of nursing students.

## 2. Materials and Methods 

**Figure 1:**
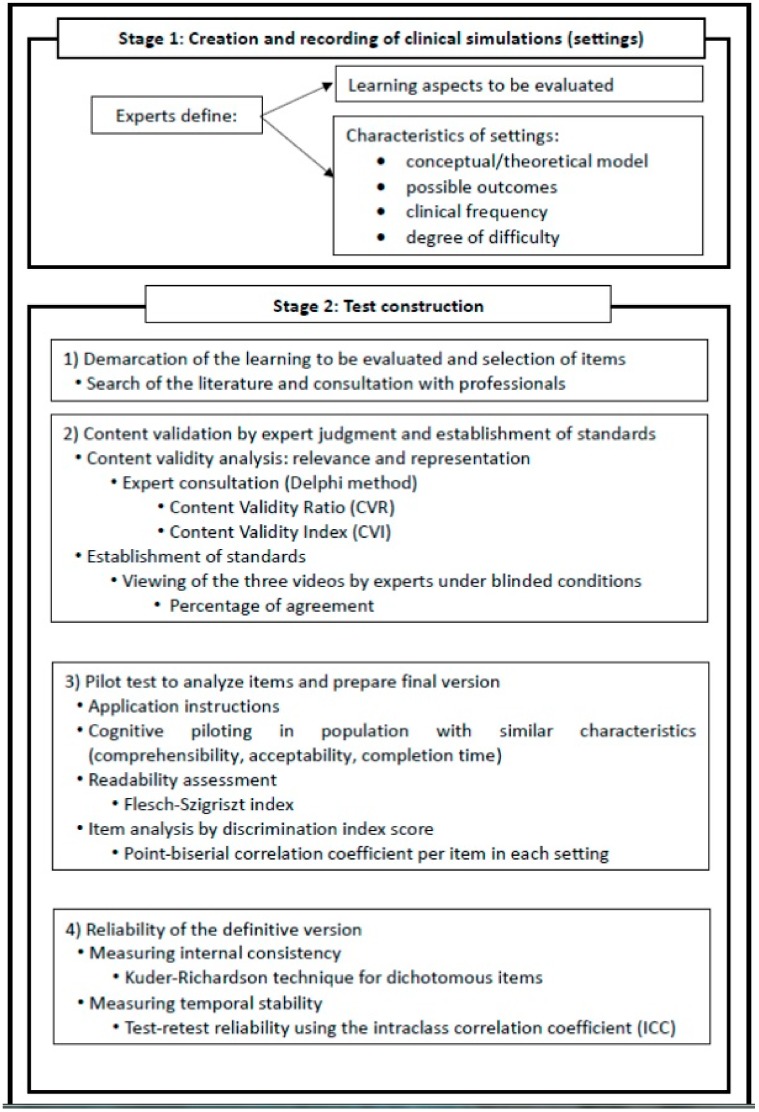
General diagram of the study: process of questionnaire development.

A two-stage study (Figure 1) was conducted between February 2017 and January 2019 to develop and then validate a CRT to evaluate BIT learning, comparing test results with standards previously established by the expert group [25]. 

Stage 1: Creation and recording of BTI simulations in different settings and the establishment of evaluation standards. The selection of experts was based on three criteria: (a) variability in their professional profiles; (b) clinical competence and experience of providing patients with anti-tobacco advice; (c) experience of training professionals on this issue. Eleven experts working in the Spanish public health service (2 physicians, 2 nurses, and 7 psychologists) were selected, all with more than 10 years’ experience of smoking-related care in a community setting. This group consensually defined the learning aspects to be evaluated, explored the application of the 5As+5Rs model, and developed and selected typical clinical settings with different outcomes and problems for video recording. 

Stage 2: The development of the CRT was similar to proposals by Hambleton and Rogers [26] and structured in the following four phases:

Phase 1: Demarcation of the learning evaluated and selection of items.

The expert group selected the theoretical model to be followed and defined behaviors that would indicate BTI learning acquisition. A search of MEDLINE, CINAHL, and SciELO databases was conducted to identify items for inclusion, using the terms “survey”, “brief intervention”, and “tobacco” and including all studies in English or Spanish up to June 2018. The final selection was then made, only including studies on instruments to evaluate BTI learning in health science professionals and/or students.

Phase 2: Content validation by expert judgment and establishment of standards. 

Based on the draft developed in phase 1, the expert group used the Delphi consensus technique to assess the adequacy, relevance, and readability of each item on a 5-point Likert scale. The final selection of items was determined according to their Content Validity Ratio (CVR > 0.70), and the Content Validity Index (CVI) was calculated for the whole instrument (CVI > 0.80). Members of the expert group were also asked for any proposals to improve the instrument. Evaluation standards were established by the experts after each had viewed the simulations in three settings and evaluated the intervention in a blinded manner, followed by the calculation of the percentage of items with identical responses from all experts. The acquired learning was scored as the percentage of correct responses according to the standard established in each setting.

Phase 3: Pilot test to analyze items and prepare final version.

Instructions for the pilot test and the scoring criteria were based on the instrument developed in phase 2. First, cognitive piloting was conducted in a group of 10 second-year nursing students to assess the comprehensibility, acceptability, and completion time for each setting. Readability was assessed with the Flesch-Szigriszt index [27] using INFLESZ software and classifying scores as follows: <40 = very difficult, 40–55 = somewhat difficult, 55–65 = normal, 65–80 = rather easy, and >80 = very easy.

Next, a pilot test was conducted in a group of 160 second-year nursing students, half with BTI training, who viewed and responded to the three videos. Second-year students were selected because it is the first year in which they would receive training related to anti-tobacco interventions in the nursing degree course of the University of Murcia. The instrument was administered under the same conditions as intended for its clinical application. Results were used to calculate the point-biserial correlation coefficient (discrimination index) per item in each setting, considering values >0.30 to be adequate.

Phase 4: Evaluation of the reliability of the definitive instrument. 

The definitive version of the test was administered to a group of 100 second-year nursing students who had not participated in the pilot tests. Its reliability was determined by using the Kuder-Richardson technique for internal consistency and the test-retest method, repeating the test on 33 participants after a 14-day interval and calculating the intraclass correlation coefficient (ICC) with 95% confidence interval (CI).

### Ethical Considerations

All participants gave their consent to participate in the study, which was approved by the Research Ethics Committee of the University of Murcia (ID: 1968/2018). 

SPSS Statistics for Windows, version 23.0. (IBM Corp, Armonk, NY, USA) was used for the data analysis, applying the tests reported above. 

## 3. Results

### 3.1. Stage 1

We recorded three videos of around 2.5 min in duration. Each video (setting) simulated a BTI involving a primary healthcare professional and patient, with different outcomes and including various pitfalls (e.g., unstructured/non-systematic BTI, failure to record clinical history, and non-personalized advice). The three possible outcomes in the 5As+5Rs model are that the patient quits smoking, is willing to make an attempt to quit smoking, or is not willing to make this attempt. Given that the first outcome is not controversial, the second outcome was simulated in settings 1 and 2 and the third in setting 3, including different pitfalls. 

### 3.2. Stage 2

#### 3.2.1. Phase 1: Demarcation of Evaluated Learning and Item Redaction 

The instrument was designed in accordance with the 5As+5Rs model [17,18]. In the literature search, 393 records were retrieved from MEDLINE, and 32 of these were selected after reading their titles and abstracts; 58 and 12 different records were retrieved from CINALH and SciELO respectively, but none were selected. After a reading of the full texts, 14 studies were finally chosen: three on instrument validation and 11 on BTI learning evaluation (Table S1). After reading these studies, 15 items were chosen and adapted to the instrument, and further items were redacted to complete the theoretical model. The instrument was organized into five sections (5As) with a total of 24 items. After viewing the videos, students responded to each dichotomous (yes/no) item on the performance of the professional.

#### 3.2.2. Phase 2: Content Validation by Expert Judgment and Establishment of Standards

After the first expert round, low CVRs were obtained for adequacy (items 4, 16, 20, and 24), relevance (items 4 and 24), and readability (items 4, 8, 12, and 24) (Figure S1). These items were re-written for the second expert round, in which they all achieved a CVR >70% except for item 24 (“The evaluation of willingness to quit smoking is repeated”), which was therefore eliminated. The final BTI-St^©^ contained 23 items and obtained a CVI of 0.84. For the procedure to establish standards, the percentage of responses on which the experts were unanimous was 94.5% for responses in settings 1 and 2, which were resolved with the response to 11 items (Figure S1: items 5–15) and 98.2% in setting 3, which was resolved with the response to 15 items (Figure S1: items 5–11, 16–23). There was disagreement between two experts on the response to item 6 in setting 1, item 10 in setting 2, and item 11 in all three settings. The experts proposed that the test should follow an algorithmic rather than linear format (Figure S1). 

#### 3.2.3. Phase 3: Pilot Test to Analyze Items and Prepare Final Version

No items were modified after assessing the results of the pilot test and its comprehensibility and acceptability. The time to complete the test was 2.5 min in each setting. The instrument obtained a Flesch-Szigriszt index score of 58.04, indicating normal readability. A discrimination index score >0.30 was achieved by all items in all three settings, indicating adequate discrimination (Table 1). 

#### 3.2.4. Phase 4: Reliability of the Definitive Version 

The test demonstrated adequate reliability, obtaining KR-20 values of 0.81 in setting 1 (11 items), 0.86 in setting 2 (11 items), and 0.88 in setting 3 (15 items), and ICC (test-retest) values of 0.73 (95% CI = 0.44–0.86; *p* < 0.001) in setting 1, 0.68 (95% CI = 0.35–0.84; *p* < 0.001) in setting 2, and 0.82 (95% CI = 0.63–0.91; *p* < 0.001) in setting 3.

## 4. Discussion

This study describes the development and validation of an instrument to evaluate BTI learning in nursing students using videos of standardized clinical situations. To our best knowledge, BTI-St^©^ is the first CRT-based instrument on BTI learning and allows the detection of learning deficits and the implementation of remedial measures. 

Although the creation and video-recording of simulations is time-consuming and requires the services of experts, this method allows performance variations to be attributed to the learning of students rather than the characteristics of cases [28]. This approach has previously been adopted in the training of nursing students, although evaluations have required the participation of experts [29] or the employment of subjective self-evaluation questionnaires [30]. 

The BTI-St© is based on the 5As+5Rs model, which has been used in most research on instruments to that evaluate the BTI learning of health care professionals and students [18,19,21,31,32,33]. The consensus of our expert group on the final version of the instrument confirms the relevance of the items selected [34]. Only one item was eliminated because it referred to repeated evaluations of the readiness to quit smoking, and all three settings simulated a first visit alone. The items obtained satisfactory discrimination values, demonstrating that the scores are valid indicators of BTI learning. Scores at the minimum threshold were obtained for Item 10 in settings 2 and 3 and for item 11 in all settings. There was also some discrepancy among the experts for item 11 (Assessing whether the person thinks they can quit smoking), probably because this aspect was less clearly shown in the videos. The internal consistency of the instrument was found to be adequate, obtaining values > 0.80 in each setting, and the test–retest temporal stability ranged between moderate (ICC of 0.68) in setting 2 and high (0.82) in setting 3. In addition, testing of the instrument in three different clinical scenarios with distinct contents further supported the reliability of its results.

The definitive version of the instrument was found to be comprehensible and acceptable by the students, and its readability is normal according to the Inflesz scale; therefore, no rewriting is needed. Furthermore, its application requires very little time, around 2.5 minutes to watch the video plus 2 minutes to respond to the questionnaire.

The WHO recommends the assessment of BTI learning for health care professionals and students [15], but this has been carried out in less than half of the numerous programs taught on nursing courses [35]. It is crucial to determine the effectiveness of learning because this allows the introduction of improvements, the comparison of different approaches, and detection of variations among distinct types of trainee [36]. 

The BTI-St© is based on the 5As+5Rs model, whereas some authors have used unconsolidated models [16,37], have only partially followed the 5As model [32,38], or have not reported the model used [39]. Our instrument also has the advantage of greater objectivity in comparison to the self-evaluation approach that has been employed in many studies [20,21,31,32,39]. Methods that depend on expert evaluations using VOSCE or OSCE rubrics have also been limited by a failure to test their validity [16,22,37,40] or their validity and reliability [33] (Table S1).

BTI-St© can be useful in various teaching and evaluation approaches, including high-fidelity clinical simulations that contain an experiential component and a reflexive element, as in post-simulation debriefing [41] and it can also complement expert OSCE and VOSCE evaluations. 

### Limitations

We did not determine the convergent validity of the BTI-St© with respect to a different independent instrument. It would also be of interest to explore the impact of an educational intervention on BTI learning. The present results were obtained in nursing undergraduates, and the validity of this instrument should also be tested in other students of health sciences. Finally, BTI-St© only evaluates the brief intervention itself. It takes no account of the communication quality or the generally long-term nature of primary care, the most frequent setting for this type of intervention.

## 5. Conclusions

BTI-St© is a robust and reliable instrument that can be simply and rapidly applied. It follows the WHO 5As+5Rs model and offers a more objective criterion-referenced evaluation of BTI learning in nursing students. BTI-St© can be used to evaluate the effectiveness of different educational interventions, facilitating the diagnosis of deficits in the learning of any of the five As.

## Figures and Tables

**Table 1:** Results of discrimination analysis of brief tobacco intervention (BTI)-St items in the three settings.

	Setting 1	Setting 2	Setting 3
(BTI)-St Items	Corrected Item—Total Correlation	Corrected Item—Total Correlation	Corrected Item—Total Correlation
Item 5 Classifying the person as a smoker	0.46	0.34	0.35
Item 6 Recording this information in their clinical history	0.48	0.40	0.69
Item 7 Clearly offering advice	0.40	0.35	0.64
Item 8 Assertively offering advice	0.55	0.45	0.64
Item 9 Offering advice in a personalized manner	0.52	0.46	0.63
Item 10 Assessing whether they would like to be a non-smoker	0.48	0.32	0.31
Item 11 Assessing whether the person thinks they can quit smoking	0.31	0.32	0.30
Item 12 Providing motivation based on the importance of quitting for the person	0.59	0.67	
Item 13 Reporting the risks of smoking for the person	0.60	0.50	
Item 14 Asking the person to identify the benefits of quitting that they consider important	0.55	0.65	
Item 15 Asking the person to identify the barriers/obstacles to quitting	0.63	0.57	
Item 16 Indicating a date to quit smoking (preferably within 2 weeks)			0.53
Item 17 Indicating the need to communicate the quitting attempt to family, workmates, and friends and to ask them for help			0.58
Item 18 Indicating the need to anticipate difficulties (situations that can hamper quitting)			0.58
Item 19 Recommending the person to make his/her house a tobacco-free environment			0.59
Item 20 Describing the availability of pharmacological treatments if appropriate			0.43
Item 21 Offering complementary materials (brochures, quit line, etc.)			0.51
Item 22 Agreeing on a follow-up contact			0.44
Item 23 Referring the person to specialized resources for quitting (if available)			0.60

## Supplementary Materials

The following are available online at https://www.mdpi.com/1660-4601/16/20/3944/s1, Figure S1: Brief Tobacco Intervention (BTI-St^©^), Table S1: Comparison of instruments to measure anti-tobacco advice.
