# Supplementary material for: Design and Validation of an Instrument to Evaluate the Learning Acquired by Nursing Students from a Brief Tobacco Intervention (BTI-St©)
Source: Int J Environ Res Public Health. 2019 Oct 16;16(20):3944. doi: 10.3390/ijerph16203944 (PMC6843560; doi:10.3390/ijerph16203944)
Supplement: Supplementary file 1 [file ijerph-16-03944-s001.pdf]

International Journal of
Environmental Research and Public Health

MDPI

Supplement

Table S1. Comparison of instruments to measure anti-tobacco advice.

Author, year	Conceptual Model	Target Population	Tool (nº items)	Administration Method	Validity Tests	Reliability Tests
Ockene, J.K. et al. 1988	Counseling skills in six content areas: (1) desire and motivation to change; (2) experience with smoking cessation; (3) barriers; (4) strengths; (5) plan for change; (6) methods of dealing with factors that may interfere	Physicians-in-training	Counseling skills scale: (1) ability to elicit information; (2) provide information; and (3) elicit and deal with patients' feelings toward smoking in the 6 content areas. (18 separate scores)	Evaluation by experts using VOSCE	No	Significant Kendall's coefficient of concordance for scores of the three experts in each skill area
Corelli, R.L. et al. 2005	5As model	Pharmacy students	Students' self-rated abilities for cessation counseling (12 items)	Self-evaluation	No	No
Kerr, S.M. et al. 2007	5As model	Under- and postgraduate students of nursing, physical therapy, occupational therapy, and podiatry	Practice subscale (10 items)	Self-evaluation	Adequate content validity	Weighted kappa ranging from 0.35 to 0.49 - ICC value for total practice scores = 0.84 (95% CI 0.8—0.88)
*Delucchi, K.L. et al. 2009	The National Cancer Institute's "4As" approach	Staff working in drug abuse treatment or HIV care	Smoking knowledge, attitudes, and practices (S-KAP) instrument (46 items). Practice subscale (8 items)	Self-evaluation	Construct validity: EFA= 5 factors	Cronbach's alpha (practice subscale) = 0.91

Sreeramareddy, C.T. et al. 2010	Not indicated	Medical students	Clinical practices on tobacco smoking habits (8 items)	Self-evaluation	Pre-tested in medical students	No
*Newhouse, RP. et al. 2011	5As model	Nurses	Smoking Cessation Counseling Scale (SCC) (24 items)	Self-evaluation	- Convergent validity $R^2 = 0.36$<br>- Construct validity: EFA = 4 factors. KMO = 0.95. 68.3% of variance explained	Cronbach's alpha = 0.95
Wadland WC. et al. 2011	5As model	Medical students	Assessment in trained, standardized patients Evaluation on a 10-point scale	Evaluation by experts using VOSCE	No	No
Molina A.J. et al. 2012	Partial 5As model	Healthcare students	Perception of ability to carry out intervention for smoking prevention and control (3 items)	Self-evaluation	No	No
Sohn, M. et al. 2012	5As model	Nursing students	Self-efficacy in performing smoking cessation intervention (9 items)	Self-evaluation	No	Cronbach's alpha = 0.86
Romito et al. 2014	5As model	Dental students	Student performance on 5As as assessed by OSCE checklist (5 items)	Evaluation by standard patient self-evaluation. Evaluation by co-workers	No	Interrater agreement of standardized patients, students, and peers: 63.0–93.6%
*Mazor, K.M. et al. 2015	5As model	Medical students	Behavioral checklist of tobacco–dependence treatment skills (33 items)	Evaluation by experts using OSCE	- Content validity by experts<br>- Pretest scoring criteria with 31 students<br>- Correlation between raters' global assessments of communication skills	Coding accuracy, only 3/64 double-coded encounters found discrepancy in 1–2 items

					and checklist- based scores = 0.42 ($p < 0.001$)	
Lucas, N.W. et al. 2016	5As model and ''ABC'' approach	Medical students	Knowledge on smoking cessation in medical students (K.I.S.S.) (10 items)	Evaluation by experts using VOSCE	No	Inter-rater variability Kappa = 0.642
Abdelazim, et al. 2018.	5As model	Family physicians	Practice of smoking cessation counseling observation checklist (10 items)	Self-evaluation	Content validity by 5 experts<br>Pilot study in 20 physicians to test clarity and applicability	Cronbach's alpha = 0.75
Hagimoto A. et al. 2018	Counseling skills in six content areas:<br>(1) desire and motivation to change; (2) experience with smoking cessation; (3) barriers; (4) strengths; (5) plan for change; (6) methods of dealing with factors that may interfere	Health professional s (physicians, registered nurses, and public health nurses	Smoking cessation counseling skills (6 items)	Evaluation by experts using VOSCE	No	Interrater reliability, ICC for total score = 0.82 ($p < 0.001$)<br>Test-retest method: $\varrho = 0.98$ ($p < 0.001$)<br>Cronbach's alpha = 0.59

International Journal of
Environmental Research and Public Health
MDPI

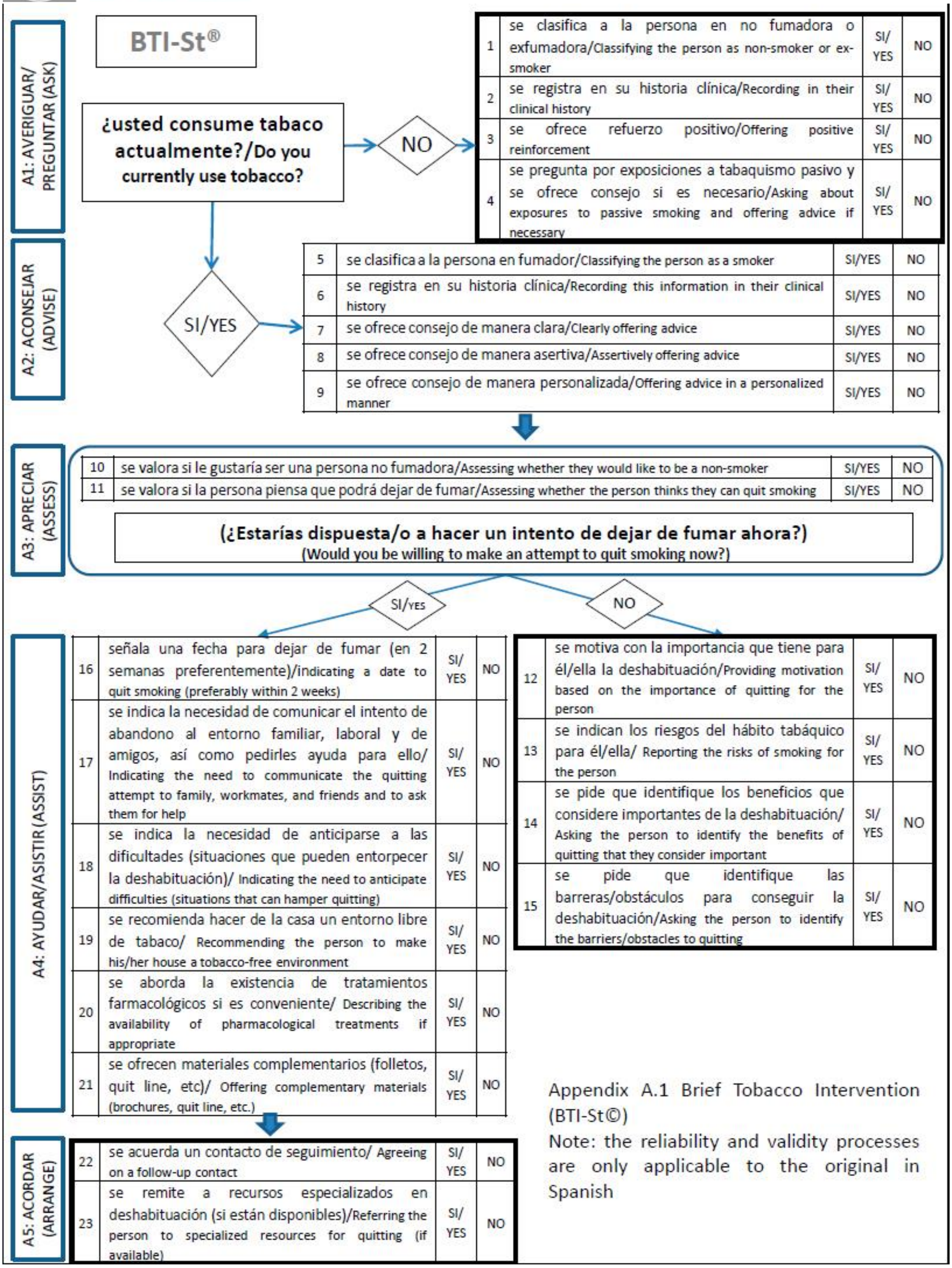

Figure S1. Brief Tobacco Intervention (BTI-St©).
